# Hints on Medical Education in London

**Published:** 1809-10

**Authors:** 


### *To the Editors of the Medical and Physical Journal.*

GENTLEMEN,

PERMIT me to occupy a page or two of your Journal, in the statement of some observations, which I have to make upon a subject of much importance to the most numerous class of the medical profession.

The time draws near, when the Winter Courses of Lectures upon medical subjects, commence at the different hospitals and private schools in the metropolis. This is the period, when the younger branches of the profession, who are just emancipated from the trammels of aprenticeship, bend their steps towards London, to "finish", as it is termed, their education. The qualifications which young men possess, at the termination of their apprenticeship, must vary, according to the advantages of their situation and education. Many there are, who under the direction and assistance of an indulgent and scientific master, have laid a happy basis on which to prosecute their future studies. Many have not possessed these advantages; for there are masters, in the medical as well as in other professions, who, as if careless of the future interests of their apprentices, are only anxious that their medicines are compounded properly, and that the business of the surgery be not neglected. If a young man, thus situated, reads, it is without method, and, probably, with little benefit. Or, if he chuses to spend his leisure hours in idleness, it passes unheeded by his master. Such a youth will but rarely afterwards prosecute his studies with success, or shine in his profession; he enters upon the practice with disgust, gust, because he feels no confidence in his acquirements.

Most of the young men, resorting to London to attend the lectures and practice of the different hospitals, arrive without having formed any previous plan, or obtained much previous information; generally speaking, they are unacquainted with persons who are capable of recommending them to the different lecturers, or of giving them any asistance in professional objects.*

The first, and not the least important affair, which engages the pupil's attention, is procuring lodgings or rooms; if he applies for information at the hospital, as is generally the case, he finds no difficulty in obtaining recommendations; many fellow pupils are ready to step forward with advice, and the stranger thinks himself happy in being able to occupy the same rooms as an old pupil, from whose society he hopes to obtain much information and comfort. Perhaps in these respects he is fortunate; his companion may be every thing he could wish or desire, steady, studious, and communicative. But if, on the contrary, he should be a rake and a libertine, his situation must be dangerous; and if he does not change his abode, the probability is, that he will ultimately fall a victim to the insidious and designing counsels of his companion, who will lose no opportunity of reducing him to the same level with himself; if he succeeds, his money and time will be spent at the theatres, billiard tables, and in idleness. He will be seen occasionally at the lecture room, but even there he will generally be found either sleeping or interrupting the more studious and attentive pupils. To study is a task too heavy for him, and he relinquishes it altogether.†

If a young man is not provided by his friends with a situation before his arrival in town, but is left to look out for himself; if he is a stranger, and has no young friends that he could depend upon, I would advise him to take rooms solely to himself, and, I am convinced, that if

Y 4 economy

* These observations, altogether, are more particularly applicable to Guy's and St. Thomas's Hospitals.

† These remarks are strictly true, though I hope they will apply but to few. Last Autumn and Winter, I knew a young man, a student at St. Thomas's, who, for four months together, might be almost constantly seen at the billiard rooms in the Borough; several times have I seen him playing for half-a-guinea a game! Before his arrival in town, which was at the beginning of October, he had never seen a billiard table!

economy is an object, this will be his best plan; he should be extremely cautious whom he admits to intimacy and friendship; they should be few in number, and those from whom he is likely to obtain information as well as pleasure. The studies necessary to qualify a surgeon-apothecary are numerous and expensive, so that a student in such a situation, ought to find but little time to waste on idle and expensive amusements. Let him not neglect to pay a proper regard to Sunday: his situation is a dangerous one for moral and religious principle, and he must learn to treat ignorance and immorality with silent contempt.*

But the most important object for consideration, is the plan which the pupil ought to adopt and follow in his studies. Many, I think I may add most, young men are sent to town with only general advice, perhaps of this kind: Remember your time will be short, therefore study all you can, and, if possible, attend all the lectures; but first enquire at the hospital, there you can gain satisfactory information, and form your judgment accordingly. The pupil follows this advice, and he is recommended, directly or indirectly, to enter himself a pupil to them all at the same time; and as an inducement to this, the lectures "collectively," if a person enters as "perpetual pupil," are cheaper than if they were entered upon "singly," (with one or two exceptions). If a pupil adopts this plan, he receives cards of admittance to Lectures on Anatomy, Surgery, Medicine, Chemistry, Midwifery, Physiology; Theory of Medicine, Therapeutics, &c. and Philosophy, and without consideration or proper advice, he at once enters upon the study of all these subjects, and thinks to master them all at the same time. To a mind the most comprehensive, this would be an unequal task, and all that the pupil can acquire, will be superficial knowledge, which will very soon be forgotten. He may enter upon his studies with ardor and enthusiasm, but such a variety of objects requiring attention, in very quick succession, will but too often create confusion and disgust. I do not mean to deny, that a man destined for the medical profession should be well versed in every department of the

* Could it be believed that young men, who a few months before left steady families in the country, could be so far lost to shame and sunk in depravity, as to spend their Sundays in card-playing and in drinking, &c. Yet such scenes as these, more than once, came to my knowledge last winter.

the studies and sciences above mentioned; they are all connected with the science of medicine. My only object is, to state, that a student, at the commencement of his studies, should begin only with two or three rudimental or fundamental branches of his profession; and as his mind enlarges, and when he clearly comprehends the elements, let him then enter upon those studies that may be necessary for completing his scientific and medical pursuits.

It is generally admitted that anatomy is the basis, the foundation upon which the superstructure of a medical and surgical education is to be erected. Few practitioners have had merited fame, or left behind them any tracts of deep knowledge in the science of medicine, but such as have evidently bestowed pains and made some progress in this essential study. This is the only real and substantial basis for comprehending, with perspicuity, the structure of parts in health, and the changes produced by disease. It is this accomplishment that conducts a professor of the art to a clear comprehension of the parts under disease or concerned in operations.

If the student, during his apprenticeship, has obtained a moderate knowledge of medicine and materia medica, perhaps he could prosecute the study of chemistry with advantage at the same time with anatomy. This science, as connected with medicine, is unquestionably of primary importance, and but few young men, during their apprenticeship, acquire much knowledge of it; for this reason, men who have been in practice some years, are generally but slightly acquainted with chemistry and the new nomenclature. They prescribe in the language of our old Pharmacopœias, and because they will not, or cannot find time, to keep pace with the rapid and important improvements in this science, they neglect them altogether. This observation will extensively apply to country practitioners; chemistry, as a science, is as yet but little cultivated among them; and I know several instances where pupils have been prevented, by their parents or masters from attending with the rest of their studies, chemical lectures, which by them were considered unprofitable and unnecessary.

When the pupil has gained a good knowledge of anatomy, he should then enter upon the study of surgery and physiology. In this study, a caution is particularly necessary; pupils are apt to direct too much of their attention to "capital operations," which should be considered as information likely to be seldom demanded, as the least creditable means of cure, and, with few exceptions, as as last resources. Before an operation is performed, the surgeon has to decide upon the propriety and necessity of it, which requires great judgment, with much practical and discriminative knowledge; and this the pupil will learn in the lecture room and in the wards of the hospital, rather than in the operating theatre. In some of the hospitals in town, operations are conducted with much parade and show, which has every imposing effect upon the mind of inexperienced youth.

The theory and practice of midwifery may be attended to at a time most convenient to the student. If he remains in town during the summer months, he will have an excellent opportunity for attending to this important part of his studies, as his attention will not be divided by other lectures.

It is obvious that punctuality in attendance to lectures is of the utmost importance in every department, and should be particularly insisted upon by the professors. Neglect and inattendance (which are but too common) should in some way be punishable; perhaps, the best means for a lecturer to insure attendance, would be, to withhold his certificate in case of irregularity. It is now the practice for lecturers to give certificates, specifying "diligent attendance, &c." to pupils indiscriminately; thus, he who has scarcely been in the lecture-room during a whole course of lectures, obtains the same honorary compliment from his preceptor, as he who has scrupulously attended every lecture.

Within these few years, it has become much more common for young surgeons to aspire to the honour of becoming "Members of the Royal College of Surgeons." If the College was established for the purpose of excluding from the profession, ignorant and unlearned men, surely their examinations should be more severe. Is it not a severe reflection upon the College, to pass a young man after asking him only three questions?* Could they, with propriety, decide from such a slight examination, that he was a capable and fit person for practising the art of surgery? I have more than once known, very young persons obtain

* A fellow pupil of mine, in the Spring of 1808, obtained a diploma, being asked only three common questions! Another was asked only six; and many have I known who have not been asked twenty. [Perhaps, in these instances the Examiners might have a personal knowledge of the candidates' acquirements.—Ed.]

obtain diplomas, before they had attended lectures or hospital practice three months! and even those who never touched a human body with a dissecting knife! The honour and dignity of the College must suffer from such remissness on the part of the Examiners. And I hope, for their interest, as well as of the profession at large, they will be more attentive to the importance of their situation.

That these hasty remarks, which were written from personal experience and observation, may be of some benefit to the profession, is the sincere wish of

A YOUNG PRACTITIONER.

*September 5, 1809.*

